# Anti-inflammatory role of 15-lipoxygenase contributes to the maintenance of skin integrity in mice

**DOI:** 10.1038/s41598-018-27221-7

**Published:** 2018-06-11

**Authors:** Sang-Nam Kim, Seun Akindehin, Hyun-Jung Kwon, Yeon-Ho Son, Abhirup Saha, Young-Suk Jung, Je-Kyung Seong, Kyung-Min Lim, Jong-Hyuk Sung, Krishna Rao Maddipati, Yun-Hee Lee

**Affiliations:** 1 College of Pharmacy, Yonsei University, Incheon, 21983 South Korea; 2 College of Pharmacy, Pusan National University, Busan, 46241 South Korea; 3 College of Veterinary Medicine, Seoul National University, Korea Mouse Phenotyping Center, Seoul, 08826 South Korea; 4 College of Pharmacy, Ewha Womans University, Seoul, 03760 South Korea; 5 Lipidomics Core Facility and department of Pathology, Wayne State University, School of Medicine, Detroit, Michigan 48202 USA

## Abstract

15-lipoxygenase is involved in the generation of specialized pro-resolving lipid mediators that play essential roles in resolution and inflammatory responses. Here, we investigated anti-inflammatory role of Alox15 in skin homeostasis. We demonstrated that knockout (KO) of Alox15 led to hair loss and disrupted the structural integrity of the dorsal skin. Alox15 KO resulted in loss of hair follicle stem cells and abnormal transition of dermal adipocytes into fibroblasts. Alox15 deficiency increased infiltration of proinflammatory macrophages and upregulated proinflammatory and necroptotic signaling in dermal adipose tissue in the dorsal skin. Lipidomic analysis revealed severe loss of resolvin D2 in the dorsal skin of Alox15 KO mice compared to wild type controls. Treatment with resolvin D2 reduced skin inflammation in Alox15 KO mice. Collectively, these results indicate that Alox15-mediated production of resolvin D2 is required to maintain skin integrity by suppressing dermal inflammation.

## Introduction

Lipid-derived signaling molecules regulate various cellular processes including cell survival, apoptosis, and metabolism^1,2^. Among lipid derived signaling molecules, specialized proresolving lipid mediators (SPM) have been identified as endogenous lipid species that can contribute to natural resolution circuits^3^. Resolvins have been identified as one of the classes belonging to SPMs and they can be generated from the metabolism of omega-3 essential fatty acids by lipoxygenase and other lipid modifying enzymes^4^. 15-Lipoxygenase (15-LOX) is responsible for the generation of resolvin D (RvD) synthesis from docosahexaenoic acid^5^. Dysregulation of skin immunity and chronic inflammation are central pathogenic mechanisms underlying skin disease, and use of resolvins has been proposed as a therapeutic strategy^6^. Though the anti-inflammatory roles of resolvins are well known^3^, the role of Alox15 (15-LOX encoding gene) deficiency in resolvin biosynthesis and skin integrity have not been fully investigated.

The skin serves as an essential physical and immunological barrier to external insults and the entry of exogenous substances and microorganisms^7^. The skin comprises multiple layers of complex structures including the epidermis and dermis. Epidermal tight junctions^8^ and unique surface lipids^9^ have been identified as structural components of the epidermal barrier function^7^. Moreover, interactions among various cell types including epidermal cells (mainly keratinocytes), stromal cells such as fibroblasts and adipocytes, and immune cells contribute to the active defense and maintenance of skin homeostasis^7^.

The adipocyte layer within the hypodermis also constitutes a significant compartment of the skin^10^. Dermal adipocytes are reported to play an important role in hair follicle activation^11^ and skin regeneration^12^. Furthermore, recent data show that dermal white adipose tissue (dWAT) mass increases in response to infection^13^ and wound healing^12^, and inhibition of the dermal adipogenesis increases susceptibility to bacterial infection^13^. Thus, the immune function of dermal adipocytes is crucial to maintain skin homeostasis^7,14^.

Here, we investigated role of Alox15 expression in skin inflammation using knockout mice. Although the skin of Alox15 null mice appeared to develop normally after birth, skin barrier defects and hair loss were observed in adult mice, with an average onset of 16 weeks. Histological analysis of Alox15 knockout mice demonstrated elevated indices of inflammation, necroptosis and differentiation of dermal adipocytes into myofibroblasts. Mechanistically, lipidomic analysis revealed a severe loss of resolving D2 (RvD2) in the dorsal skin of Alox15 KO mice and treatment of these mice with RvD2 largely reversed the inflammatory phenotype. Our results indicate that Alox15 is required for the production of RvD2, which maintains skin integrity and suppresses inflammation.

## Results

### Alox15 expression is localized in keratinocytes and dermal adipose tissue of dorsal skin

**Figure 1:**
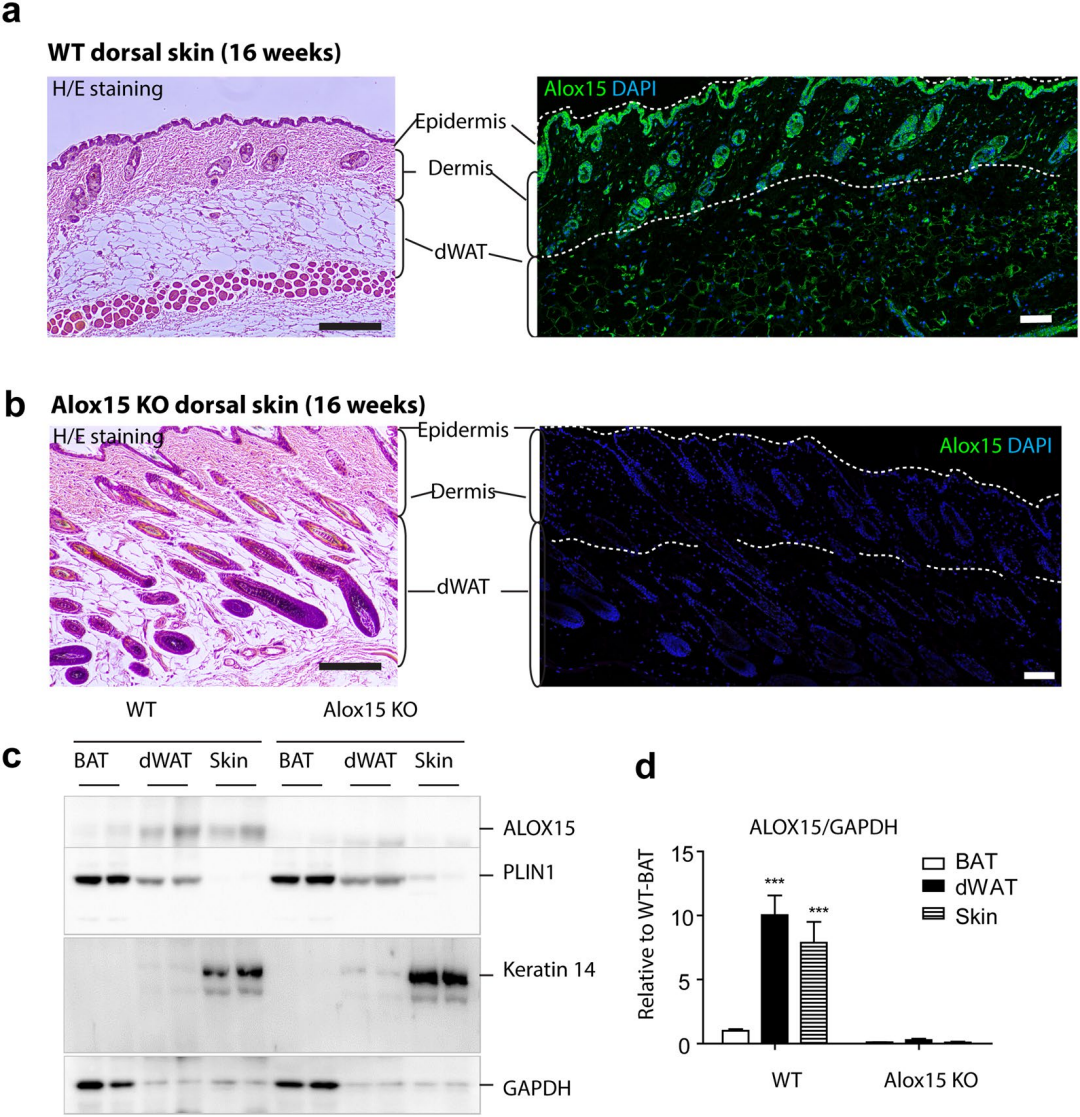
Alox15 expression in keratinocytes and dermal adipose tissue of dorsal skin of mice (**a,b**). H/E staining and immunofluorescence staining of Alox15 in paraffin sections of dorsal skin of WT mice (**a**) and Alox15 KO mice (**b**). Nuclei were counterstained with DAPI. Bar = 100 μm (**c,d**). Immunoblot analysis and quantification of Alox15 expression in brown adipose tissue (BAT), dermal white adipose tissue (dWAT) and dorsal skin of WT and Alox15 KO mice. (mean ± SEM; n = 4, ^***^p < 0.001) (Full-length blots in Fig. S1).

To investigate the role of Alox15 in skin phenotypes, we first examined the expression level of Alox15 in the dorsal skin of mice. Histological analysis demonstrated expression of Alox15 in epidermal/hair follicle keratinocytes and dermal adipose tissue (Fig. 1a). Alox15 was undetectable by immunofluorescence in all dermal layers of Alox15 null mice (Fig. 1b). For further confirmation, we performed immunoblot analysis of Alox15 from dissected dorsal skin and dermal adipose tissue layers, and brown adipose tissue. Perilipin 1 and Keratin 14 were used for adipocyte- and skin- specific expression markers, respectively. Immunoblot analysis demonstrated higher levels of Alox15 in dorsal skin and dermal adipose layers than in brown adipose tissue (Fig. 1c,d), whereas the distribution Keratin 14 and Perilipin 1 confirmed the precision of tissue dissection used in gene expression profiling experiments.

### Alox15 KO mice demonstrated hair loss and reduction in hair follicle stem cells

**Figure 2:**
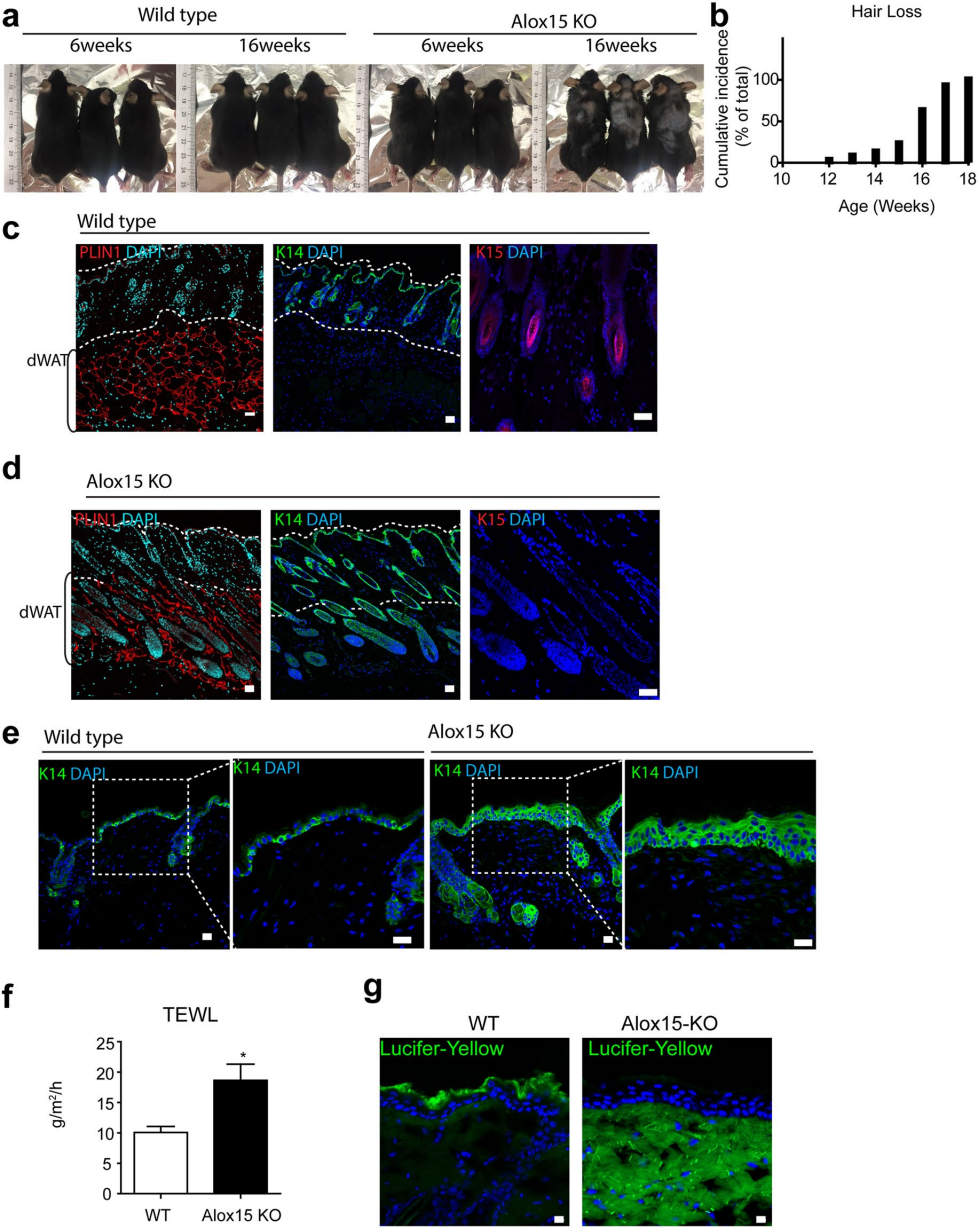
Hair loss in the dorsal skin of Alox15 knockout mice (**a**,**b**). Hair loss of the dorsal skin of Alox15 KO mice. (**b**) represents cumulative incidence of hair loss (n = 20). (**c,d**). H/E staining and immuno-fluorescence staining of PLIN1, Keratin 14 and Keratin 15 in paraffin sections of dorsal skin of WT mice (**c**) and Alox15 KO mice (**d**) (**e**) Immuno-fluorescence staining of Keratin 14 in paraffin sections of dorsal skin of WT and Alox15KO mice, along with magnified views of boxed regions. (**e**) Transepidermal water loss (TEWL) analysis of the dorsal skin of WT and Alox15 KO mice (mean ± SEM; n = 4, ^*^p < 0.05). (**f**) Lucifer yellow penetration assay of the dorsal skin of WT and Alox15 KO mice. Nuclei were counterstained with DAPI. Bars = 20 μm.

**Figure 3:**
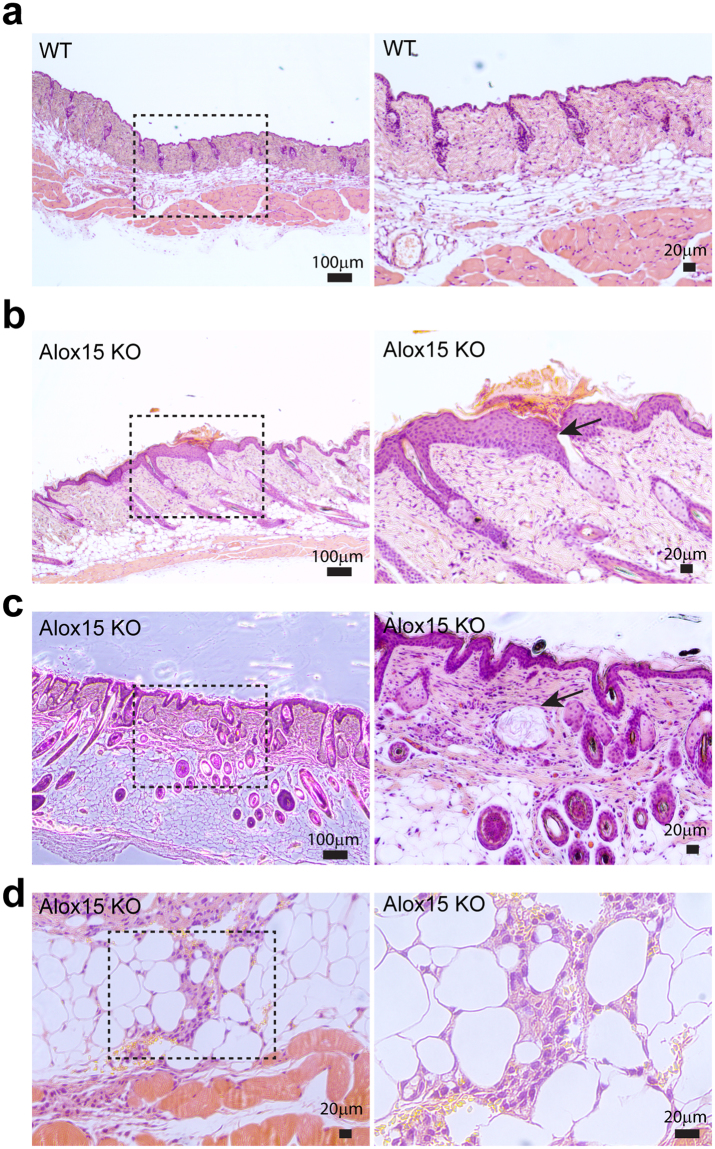
Histological analysis of the dorsal skin of Alox15 KO and wild type mice. H/E staining of paraffin sections of dorsal skin of WT mice (**a**) and Alox15 KO mice (**b–d**). Alox15 KO demonstrated thickened epidermal layers (**b**), appearance of irregular cysts (arrow) in dermis (**c**), and recruitment of myeloid cells in dermal adipose tissue (**d**) Bars = 20 μm or 100 μm as indicated.

The skin of Alox15 null mice developed normally after birth (Fig. S2); however, hair loss was observed in adult mice, with an average onset of 16 weeks (Fig. 2a,b, hair loss >1 cm^2^ area, n = 20). Immunostaining for a hair follicle stem cell marker, keratin 15, showed that the keratin 15^+^ hair follicle stem cells were reduced in Alox15 KO mice (Fig. 2c,d). while keratin 14^+^ keratinocytes were more frequently observed in Alox15 KO mice (Fig. 2c,d). In addition, Keratin 14 expression extended to epidermal suprabasal layers in Alox15 KO mice, whereas its expression was restricted in epidermal basal layers of WT control mice (Fig. 2e). Moreover, transepidermal water loss (TEWL) values were increased in Alox15 KO mice (Fig. 3f) and Lucifer yellow dye penetration was prominent in the dorsal skin of Alox15 KO mice (Fig. 3g). Further histological analysis (Fig. 3) revealed that abnormal skin structure, such as thickened epidermal layers (Fig. 3b), appearance of irregular cysts in dermis (Fig. 3c), and recruitment of myeloid cells in dermal adipose tissue layer (Fig. 3d). These data suggest that deletion of Alox15 expression resulted in loss of skin layer integrity.

### Alox15 KO showed increased inflammation in the dermis and dermal adipose tissue

**Figure 4:**
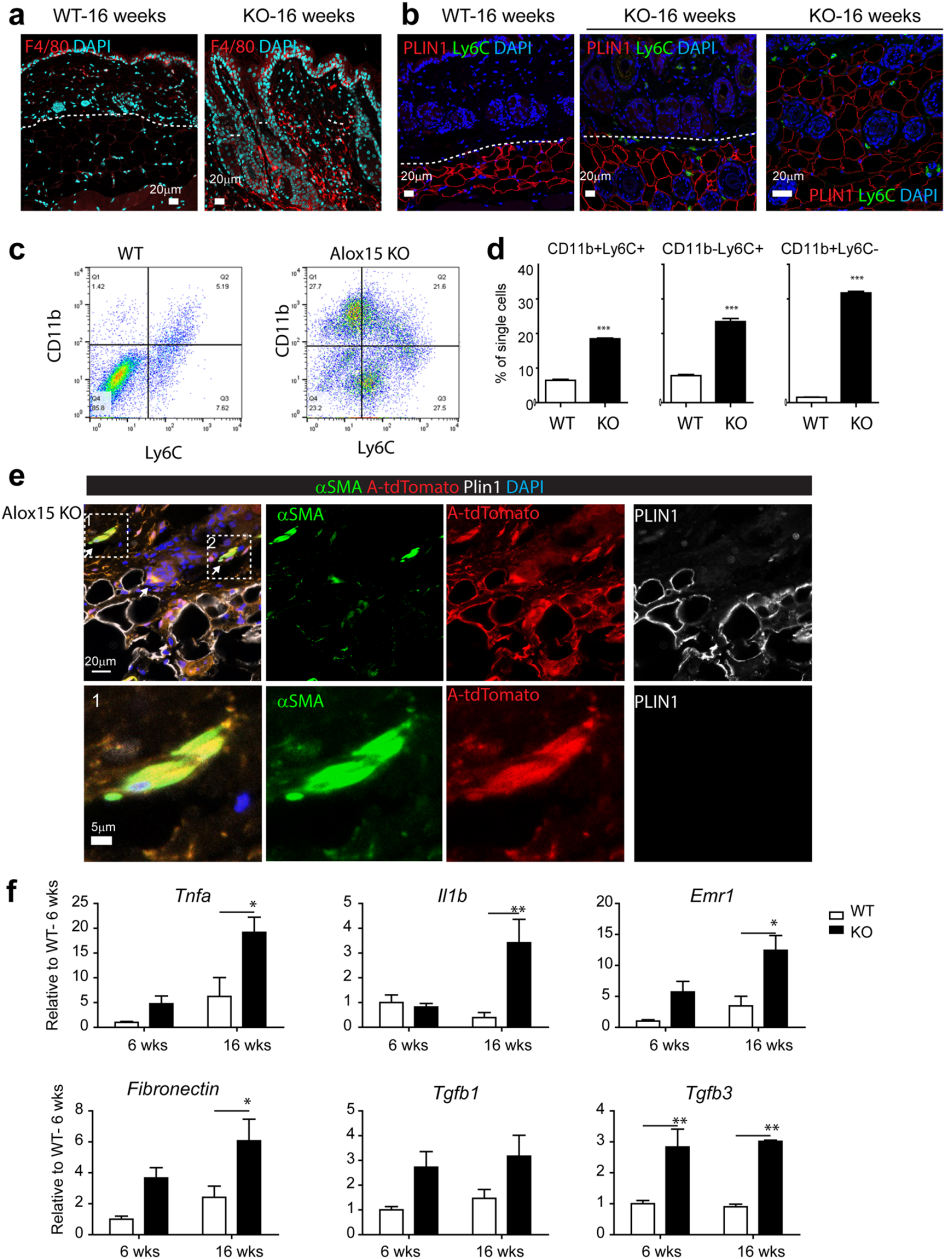
Alox15 KO showed increased inflammation and fibrogenic marker expression in the dermis and dermal adipose tissue. (**a**) Immuno-fluorescence staining of F4/80 in paraffin sections of dorsal skin of WT mice and Alox15 KO mice. (**b)** Double staining for Plin1 and Ly6C in paraffin sections of dorsal skin of WT mice and Alox15 KO mice. Right-hand side panel is shown for a higher magnification view in the dermal adipose tissue layer of Alox15 KO mice. Nuclei were counterstained with DAPI. Bars = 20 μm (**c**) Flow cytometric analysis of CD11b and Ly6C expressing cells in the dorsal skin from WT and KO mice (16 weeks). (**d**) Quantification of the flow cytometric data. (**e**) Triple staining for SMA, perilipin (PLIN1) and tdTomato in paraffin sections of dorsal skin of Alox15 KO-AdipoqCreER-tdTomato mice. Bars = 20 μm (**f**) qPCR analysis of inflammatory and fibrosis marker expression in the dorsal skin of WT and Alox15 KO mice (mean ± SEM; n = 4, Significant differences between WT and Alox15 KO were determined by post hoc pairwise comparison with Bonferroni correction. ^*^p < 0.05, ^**^p < 0.01). (See Fig. S3).

Inflammation is one of the major mechanisms underlying skin disease and Alox15 is known to be involved in generating anti-inflammatory and pro-resolving lipid mediators. Immunohistochemical analysis indicated knockout of Alox15 increased the number of F4/80+ macrophages (Fig. 4a) and Ly6C+ proinflammatory monocytes (Fig. 4b) in the dermal adipose tissues of dorsal skin. Flow cytometric analysis also indicated higher infiltration of CD11b+ and Ly6C+ myeloid cells in dermis and dermal adipose tissue (Fig. 4c,d), which have been shown to be essential in chronic inflammatory skin diseases such as psoriasis^15,16^. Because dermal adipocytes express high levels of Alox15, we hypothesized that knockout of Alox15 expression may affect integrity of dermal adipose tissue layer. Furthermore, previous studies reported that trans-differentiation of adipocytes into myofibroblasts are involved in skin diseases^14,17^. Thus, we examined the fate of adipocytes in the dorsal skin of Alox15 KO mice (Fig. 4e), using the AdipoqCreER/Rosa26-loxP-stop-loxP-tdTomato system to genetically trace adipocytes^18^. In this system, adipocytes expressing *Adipoq* are permanently labeled with the red fluorescent protein tdTomato following tamoxifen treatment. In WT controls, tdTomato expression was detected only in dermal adipocytes and not in myofibroblasts identified by alpha-smooth muscle actin immunoreactivity (Fig. S2). However, we observed tdTomato+ cells were also positive for alpha-smooth muscle actin in the skin of Alox15 KO mice, indicating the transdifferentiation of adipocytes into myofibroblasts (Fig. 4e). These double-positive fibroblasts surrounded hair follicle in dermal adipose layers. Expression profiling of dorsal skin of Alox15 mice confirmed increased expression of proinflammatory (Tnfa, IL1β, Emr1) and fibrogenic markers (Fibronectin, Tgfb3) (Fig. 4f).

### Alox15 KO showed increased necroptosis in the dermis and dermal adipose tissue

**Figure 5:**
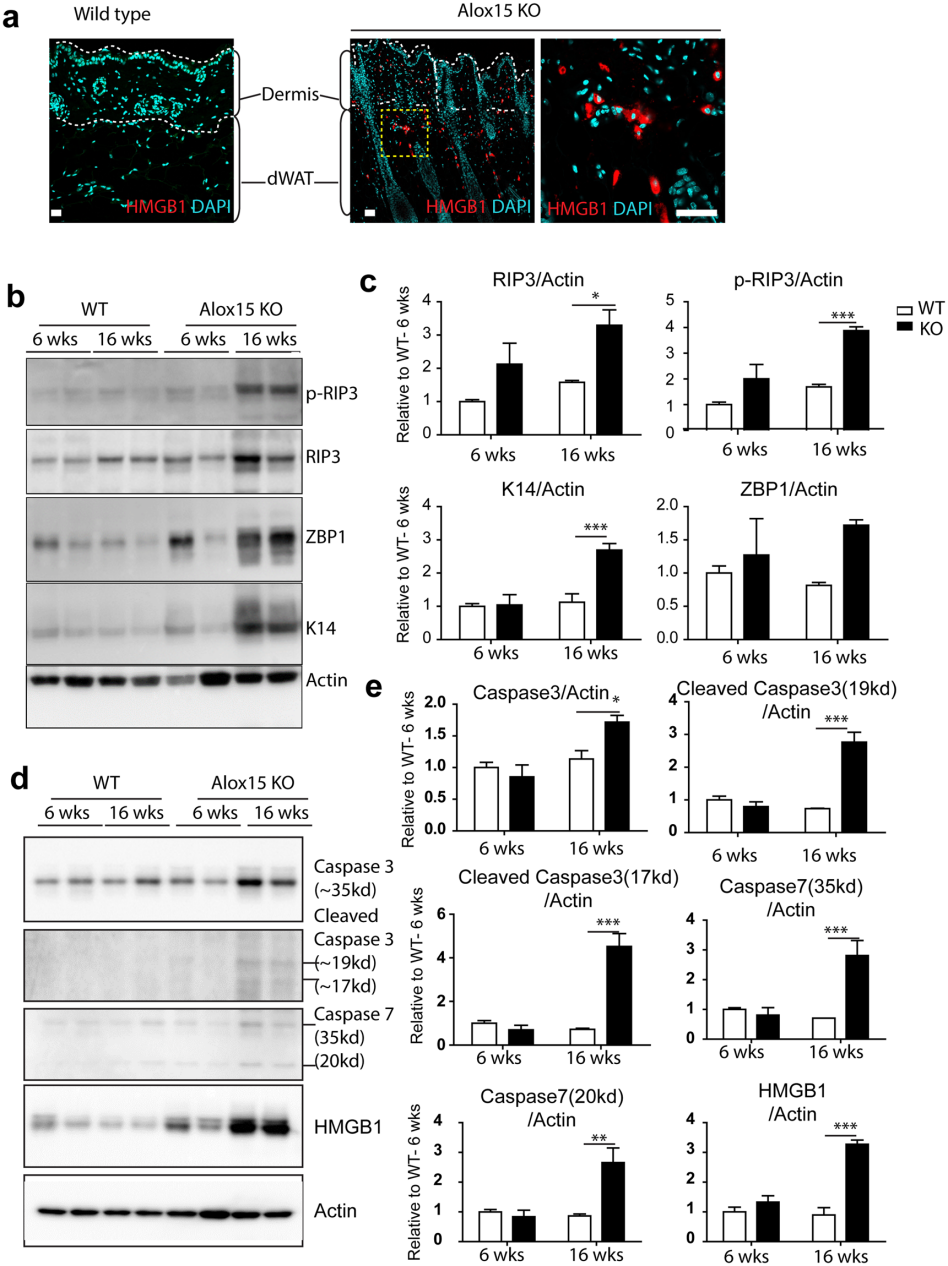
Alox15 KO showed increased necroptosis in the dermis and dermal adipose tissue. (**a**) Immunohistochemistry of HMGB1 in paraffin sections of dorsal skin of WT mice and Alox15 KO mice. Nuclei were counterstained with DAPI. Bars = 50 μm. (**b**) Immunoblot analysis of p-RIP3, RIP3, and ZBP1 expression in the dorsal skin of WT and Alox15KO mice. Actin was used as loading controls. (**c**) Quantification of immunoblot analysis (mean ± SEM; n = 4, Significant differences between WT and Alox15 KO were determined by post hoc pairwise comparison with Bonferroni correction ^*^p < 0.05, ^***^p < 0.001). (Full-length blots in Fig. S4). (**d**) Immunoblot analysis of caspase3, caspase 7 and HMGB1 expression in the dorsal skin of WT and Alox15KO mice. Actin was used as loading controls. (**e**) Quantification of immunoblot analysis (mean ± SEM; n = 4, Significant differences between WT and Alox15 KO were determined by post hoc pairwise comparison with Bonferroni correction ^*^p < 0.05, ^**^p < 0.01, ^***^p < 0.001). (Full-length blots in Fig. S5).

Because sterile inflammation can lead to cell dysfunction and death, we assessed the expression of necroptosis markers. A cell death regulator, high-mobility group box 1 protein (HMGB1)^19^ is a nuclear protein whose translocation to the cytoplasm and release from cells is a prototypic signal of cell damage^20^. Consistent with necroptosis, we observed a higher frequency of cytoplasmic HMGB1 expression in dermal cells of Alox15 KO mice (Fig. 5a). Immunoblot analysis demonstrated that phosphorylation of receptor-interacting serine/threonine-protein kinase 3 (RIP3) was elevated in Alox15KO mice, as was expression of Z-DNA binding protein 1 (ZBP1)(Fig. 5b,c)^21^. In addition, apoptosis markers (i.e. caspase 3 and caspase 7) were upregulated in Alox15KO mice (Fig. 5d,e). Together, these observations indicate increased necroptosis in the dorsal skin of Alox15 KO mice.

### Treatment with a pro-resolving species, RvD2, restores the inflammatory phenotype in Alox15 KO mouse skin

**Figure 6:**
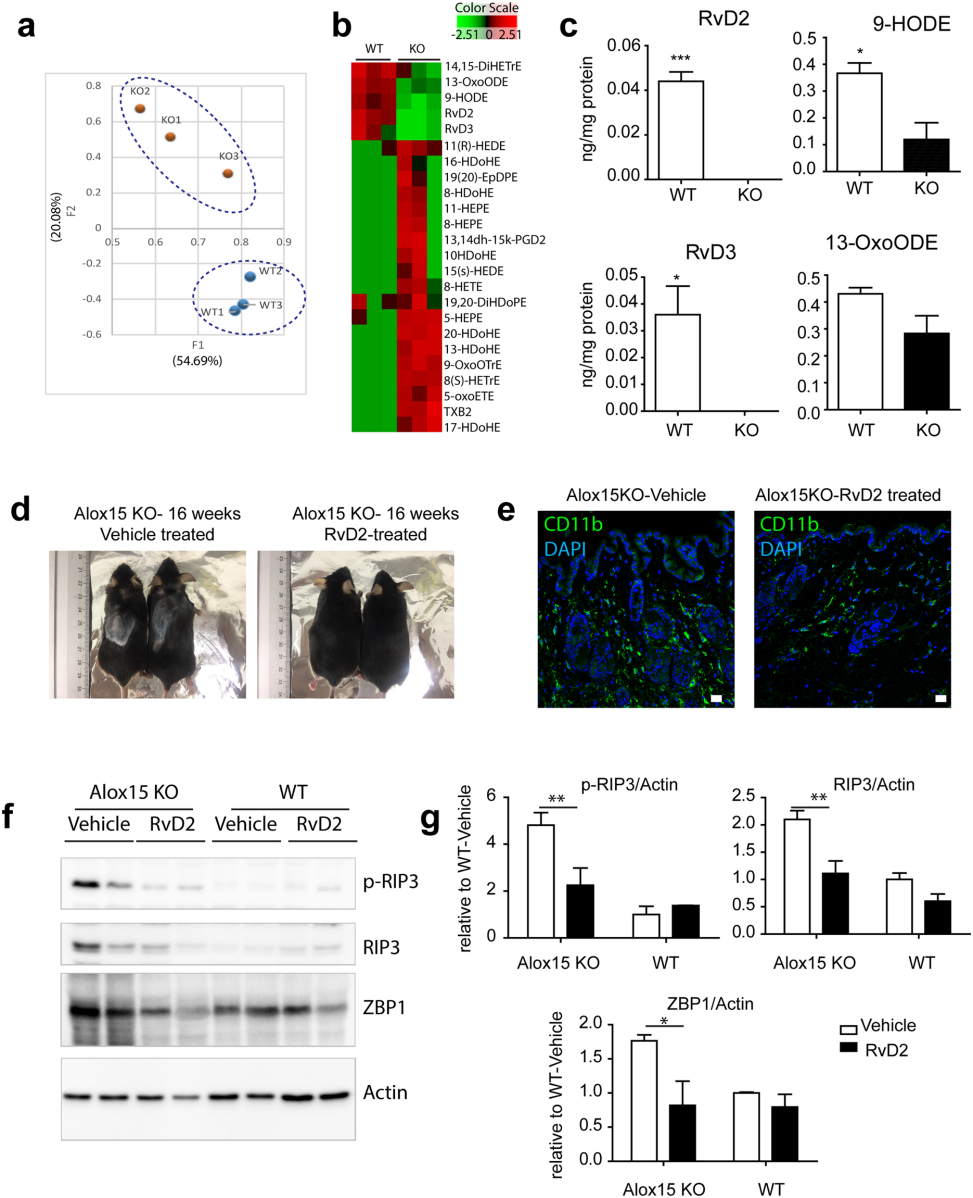
Treatment with a pro-resolving species, RvD2, restores the inflammatory phenotype in Alox15 KO mouse skin. (**a**) PCA analysis of the eicosanoids and derivatives in the dorsal skin of ALox15 KO mice and WT controls. The first two PCA components is shown. Distinct clusters of WT and KO were distinguished by component 2. (**b**) Heat map of 24 lipid species differentially regulated in dorsal skin of Alox15 KO mice compared to WT controls. (**c**) Levels of RvD2, RvD3, 9-HODE, and 13-OxoHODE in dorsal skin of Alox15 KO and WT mice (LC-MS/MS spectra and LC-chromatograms in Fig. S6). (**d**) Restoration of hair loss of Alox15 KO mice by treatment with RvD2. (**e**) Immunofluorescence staining of CD11b in paraffin sections of dorsal skin of Alox15 KO mice treated with RvD2 and vehicle controls. Bars = 20 μm. (**f**) Immunoblot analysis of necroptosis marker expression in dorsal skin of Alox15 KO and WT mice treated with RvD2 and vehicle treated controls. (**g**) Quantification of the immunoblot analysis (mean ± SEM; n = 4, Significant differences between WT and Alox15 KO were determined by post hoc pairwise comparison with Bonferroni correction ^*^p < 0.05, ^**^p < 0.01). (Full-length blots in Fig. S7).

A central function of Alox15 is the generation of bioactive oxygenated lipid mediators of arachidonic acids and other fatty acids^3^, including pro-resolving species. Therefore, we hypothesized that the metabolism of lipid by Alox15 might be crucial to maintain skin integrity and prevent pro-inflammatory phenotype. To address this hypothesis, eicosanomic profiling, including resolvins, was performed by LC-MS/MS analysis. LC-MS/MS lipidomics analysis^22^ of dorsal skin detected 90 lipid species, and principle component analysis (PCA) of these species indicated a distinct lipid metabolite profiles of Alox15 mice (Fig. 6a). Lipidome profiles were separated along the second component factor (F2) in PCA, which accounted for 21.47% of the overall variance (Fig. 6a). Clustering analysis demonstrated distinct lipid profiles of the skin from WT and Alox15 KO mice (Fig. 6a). RvD2 and 9-HODE were identified as major factors that account for the variance within the overall data set, having the highest loading scores for F2 (squared cosines of the observations: 9-HODE = 0.930, RvD2 = 0.930). Importantly, the levels of these lipid species were significantly reduced in the dorsal skin of Alox15 KO mice (Fig. 6b,c).

To determine whether RvD2 can rescue dermal defects in Alox15 KO mice, we treated the Alox15 KO mice with RvD2. As shown in Fig. 6, treatment of Alox15 KO mice with RvD2 partially restored the hair loss (Fig. 6d), reduced CD11b+ myeloid cells infiltration (Fig. 6e), and suppressed necroptotic responses (Fig. 6f,g) in Alox15 null mice, compared to vehicle-treated control mice

## Discussion

Our results demonstrate that Alox15 is critical for long term maintenance of dermal integrity. Prominent features of Alox15 KO mice were activation of pro-inflammatory signaling and great reduction in the number of K15^+^ hair follicular stem cells, suggesting loss of stemness. In addition, Keratin 14 expression extended to epidermal suprabasal layers of Alox15 KO mice, suggesting hyperproliferative characteristics of inflammatory skin conditions^23,24^. Lineage tracing of *Adipoq*-expressing adipocytes demonstrated the conversion of adipocytes into myofibroblasts, and dermal adipose layers were found to be disintegrated with increased levels of necroptosis marker expression. These data collectively suggest that chronic inflammatory milieu induced by Alox15 deficiency impaired the multi-layer structural integrity of the skin.

Although the exact mechanisms and sequential events involved in this pathogenic skin phenotype were not addressed in this study, we speculate that proinflammatory environment produced in the absence of Alox15 products contribute to defective hair follicular stem cell niches and disrupted dermal integrity. In this regard, the current study showed that Alox15 is expressed in the dermal adipocytes in skin, and thus inflammation in adipose layers could trigger initiation of a chronic inflammatory response and development of hair follicle defects. In support of our hypothesis, Alox15 deficiency resulted in epidermal hyperproliferation, which is a pathologic characteristics of chronic inflammatory skin disease, such as psoriasis^23,24^. Epidermal proliferation and thickening can be exacerbated by sustained inflammatory reaction of the epidermis and dermis, consequently leading to loss of skin barrier function and disruption of its integrity^23,24^. Loss of skin barrier function in Alox15 KO mice was evidenced by an increase in transepidermal water loss (TEWL). In addition, it is likely that proinflammatory signals from Alox15-deficient dermal adipocytes resulted in unusual transition of cell types, and impaired hair follicle cycles.

Lipidomic analysis of the skin indicated prominent reduction of RvD2 in Alox15 KO mice. Importantly, administration of RvD2 to Alox15 KO mice relieved the inflammation and partially restored skin integrity in the absence of Alox15. These data indicate that generation of resolving species by Alox15 is required for maintenance of dermal integrity. This finding might be used to develop novel therapeutic targets for inflammatory skin disease. Indeed, pro-resolving species can control inflammation in various tissues and inflammatory diseases^5^, and the potential therapeutic application of lipid mediators are increasingly recognized^3,25^. Although the RvD2 treatment was effective to reduce inflammatory responses in the skin of Alox15 KO mice, therapeutic concentration of RvD2 in the skin has not been determined in this study. Further investigations on pharmacokinetic profiles including skin concentration of RvD2 will facilitate development of resolvin-based dermatology therapeutics.

In addition to the contribution of immune cells to skin immune homeostasis, accumulated data suggest that crosstalk among keratinocytes and stromal cells are important in the regulation of skin inflammation. Based on our results, dermal adipocytes also participate in maintaining dermal homeostasis through the generation anti-inflammatory factors. While loss of RvD2 was identified as one of the triggering events that lead to chronic inflammation, other molecular players^10,13^ in the interplay between adipocytes and dermal cell types deserve further investigation^7^.

In summary, we demonstrated that Alox15 is highly expressed in keratinocytes and dermal adipocytes and is required for the maintenance of skin homeostasis. Alox15 generates anti-inflammatory resolvins, including RvD2, that are critical for long term maintenance of dermal integrity. Thus, these data suggest the potential for Alox15 metabolic pathways as therapeutics targets for chronic inflammatory skin diseases. Dermal adipocyte lipid metabolism deserves further investigation as an important cellular component that communicates with immunity network of the skin.

## Methods

### Animals

All animal experiments were conducted in strict compliance with the guidelines for humane care and use of laboratory animals specified by the Ministry of Food and Drug Safety. All animal protocols were approved by the Institutional Animal Care and Use Committees at Yonsei University. Mice were fed a standard chow diet and were housed at 22 °C and maintained on a 12-h light/12-h dark cycle with free access to food and water at all time. Male mice were used for experiments. Alox15 KO mice (JAX Mice Stock # 002778, Bar Harbor, ME, USA), were purchased and bred as previsouly described^22^. C57BL/6 mice (5–6-weeks old, male) were purchased from Orient Bio (Gyeonggi-Do, South) and used for wild type controls. AdipoqCreER (JAX Mice Stock # 024671: B6.129-Tg (Adipoq-cre/Esr1*)1Evdr/J) and Rosa26-loxP-stop-loxP-tdTomato mice (JAX MICE Stock #007914: B6.Cg-Gt(ROSA)26Sortm9(CAG-tdTomato) Hze/J) were purchased, and crossed with Alox15^−/−^KO mice to generate Alox15^−/−^adipoqCreER_tdTomato triple transgenic mice. For Cre recombination, 5 week old mice were treated with tamoxifen dissolved in sunflower oil (Sigma, 50 mg/kg body weight) by oral gavage on each of the 5 consecutive days. Alox15^+/+^(WT)/adipoqCreER_tdTomato mice were used as WT controls. tdTomato reporter expression in adipocytes was confirmed 10 days after the first dose of tamoxifen treatment, by double staining of perilipin1(PLIN1) and tdTomato on paraffin sections of dermal adipose tissue of WT and Alox15KO-adipoqCreER_tdTomato mice. For the treatment with RvD2 (Cayman), 8 week old WT and Alox15 KO mice were treated every alternate-day with RvD2 (25 ng/kg, 0.05% ethanol in saline) intraperitoneal injection for 2 weeks^26^.

### Skin barrier assays

Detection of dorsal skin transepidermal water loss (TEWL) was performed with VAPOMETER (#SWL4540, Delfin Technologies Ltd.), as previouly described^27,28^.

Skin permeability assay was performed using Lucifer yellow dye (Thermo Fisher Scientific) as previously described^27,28^. Briefly, Lucifer yellow dye in PBS (1 mM) was applied on the dorsal skin sampled from mice and the treated skin was incubated for 1 h at 37 °C. Frozen tissues were sectioned (10 μm), counterstained with DAPI (Sigma) and visualized by confocal microscopy (Zeiss, LSM710).

### Gene expression analysis

RNA was extracted using the TRIzol® reagent (Invitrogen, Carlsbad, CA, USA), and 1 μg of RNA was reverse transcribed using a cDNA synthesis kit (High-capacity cDNA Reverse Transcription kit; Applied Biosystems, Foster City, CA, USA). One hundred nanograms of cDNA was subjected to quantitative polymerase chain reaction (qPCR) in 20-μL reaction volumes (iQ SYBR Green Supermix;Bio-Rad, Hercules, CA, USA) with 100 nM primers. qRT-PCR was performed using SYBR Green dye and CFX Connect Real-time system (Bio-Rad, Hercules, CA, USA) for 45 cycles and fold change for all samples was calculated as previously described^22^. Peptidylprolyl Isomerase A (PPIA) was used as a housekeeping gene for mRNA expression analysis. Primers used for qRT-PCR were described previously^22^.

### Western blot analysis

Protein extracts were prepared as previously described^22^. Overall, 10 μg of samples were electrophoresed and blotted to PVDF membrane (Bio-Rad, Hercules, CA, USA). Western blot was performed using primary antibodies against 15-lipoxygenase-1 (Mouse, Abcam, Cambridge, UK), PLIN1 (Rabbit, Cell Signaling, Danvers, MA, USA), Keratin 14 (Rabbit, Cell Signaling), phospho-RIP3(Rabbit, Abcam), RIP3(Rabbit, Cell signaling), ZBP1 (Rabbit, Cell Signaling), cleaved Caspase-3 (Asp175) (Rabbit, Cell Signaling), Caspase 3(Rabbit, Cell Signaling), Caspase 7(Rabbit, Cell Signaling), Tubulin (Cell Signaling), and β-actin (Mouse, Santa Cruz Biotechnology, Dallas, TX, USA) and secondary anti-mouse and anti-rabbit horse-radish peroxidase antibodies (Cell Signaling) as described previously. The blots were visualized with SuperSignal West Dura Substrate (ThermoFisher Scientific).

### Flow cytometry analysis

Collagenase-dissociated cell fractions were prepared from dorsal skin of mice. For flow cytometric analysis, total 500,000 dissociated skin cells were processed for cell-surface marker staining using anti-F4/80- conjugated with Alexa Fluor 488 or APC anti-CD11b conjugated with fluorescein isothiocyanate (FITC), and anti-Ly6C conjugated with Alexa Fluor 488 or APC, (rat, 1:200, Biolegend, San Diego, CA, USA). 30,000 events were analyzed out of 500,000 cells/500ul PBS. BD FACSAria III (BD Biosciences, San Jose, CA, USA) flow cytometer was used for flow cytometric analysis. Raw data were processed using FlowJo software (Tree Star, Ashland, OR, USA).

### Tissue processing and histology

Dorsal skin of mice was processed for histological sections, and 5-μm-thick paraffin sections were subjected to H/E staining or immunohistochemical analysis. The antibodies used for immunochemical detection were anti-15-lipoxygenase-1 antibody (Abcam), Perilipin A (Cell Signaling), Keratin 14 (Cell Signaling), Keratin 15 (Cell Signaling), PDGFRA (R&D systems), smooth muscle actin-FICT (Sigma), HMGB1 (Cell Signaling), tdTomato (Clonetech), Ly6C (Biolegend) and F4/80 antibody (AbD Serotec). The secondary antibodies used were donkey anti-mouse-Alexa Fluor 488, donkey anti-rabbit-Alexa Fluor 594/Alexa Fluor 488, donkey anti-goat-Alexa Fluor 594, and donkey anti-rat-Alexa Fluor 594 (ThermoFisher Scientific, Molecular Probes). The omission of primary antibody or normal rabbit, rat, goat, or mouse IgG controls (Santa Cruz Biotechnology) was used as a negative control. DAPI (Sigma) was used for nuclear counterstaining. Cells were imaged on a Zeiss confocal laser-scanning microscope (LSM 710 META, Zeiss, Jena, Germany).

### Lipidomics analysis

Tissue samples were homogenized by probe sonication on ice (3 × 10 s). The homogenates were supplemented with a mixture of internal standards (PGE1-d_4_, RvD1-d_5_, LTB4-d_4_, and 15-HETE-d_8_, 5 ng each), extracted, and the lipid extracts were subjected to LC–MS-based lipidomic analysis to determine fatty acyl lipidome according to the standard method described previously^22,29^. Briefly, the samples were diluted with 15% methanol in water and purified on C18 solid-phase extraction cartridges (Strata-X; Phenomenex, Torrance, CA, USA). The cartridge was eluted with 0.5 ml methanol containing 0.1% formic acid directly into LC-MS autosampler vials. The eluate was dried under nitrogen, and the residue was reconstituted with 25 μl methanol. Lipid extraction and liquid chromatography tandem-mass spectrometry (LC-MS/MS) analysis were performed at the Lipidomics Core Facility of the Pathology Department at Wayne State University. Resolvin D2 ELISA Kit (Cayman Chemical) was also used to measure RvD2 levels in the skin samples according to the manufacture’s instruction.

### Statistical analysis

Statistical analyses were performed with GraphPad Prism 5 software (GraphPad Software, La Jolla, CA, USA). Data are presented as mean ± S.E.M. Statistical significance between two groups was determined by the unpaired t-test. Comparison among multiple groups was performed using a two-way ANOVA, with Bonferroni post hoc tests to determine the relevant P-values. PCA were performed with XLStat software (Addinsoft, New York, NY) to detect the common variations between variables and to visualize clusters of correlated observations. Heatmap was generated by PermutMatrix program with Euclidean distance for dissimilarity and complete linkage for aggregation criteria as previously described^30^. The graphical representation is based on Z-scores.

## Electronic supplementary material

Supplemental Figures
