# Supplementary material for: Anti-inflammatory role of 15-lipoxygenase contributes to the maintenance of skin integrity in mice
Source: Sci Rep. 2018 Jun 11;8:8856. doi: 10.1038/s41598-018-27221-7 (PMC5995961; doi:10.1038/s41598-018-27221-7)
Supplement: Supplementary file 1 — Supplemental Figures [file 41598_2018_27221_MOESM1_ESM.pdf]

Title: Anti-inflammatory role of 15-lipoxygenase contributes to the maintenance of skin integrity in mice

Authors/Affiliations: Sang-Nam Kim[1#], Seun Akindehin[1#], Hyun-Jung Kwon[1#], Yeon-Ho Son[1], Abhirup Saha[1], Young-Suk Jung[2], Je-Kyung Seong[3], Kyung-Min Im[4], Jong-Hyuk Sung[1], Krishna Rao Maddipati[5], and Yun-Hee Lee[1]*

[#]Authors are equally contributed to this work.

***Corresponding author**

Yun-Hee Lee, PhD
College of Pharmacy, Yonsei University
310 Veritas Hall D, 85 Songdogwahak-ro, Yeonsu-gu, Incheon, 21983, Korea
e-mail address: yunhee.lee@yonsei.ac.kr
Tel: 82-32-749-4522
Fax: 82-32-749-4105

Supplemental information

Figure S1. Full-length blots used in Figure 1

Figure S2. Histological analysis of the dorsal skin of Alox15 KO mice and wild type mice.

Figure S3. Lineage tracing study of Alox15 KO-AdipoqCre-tdTomato mice related to Figure 4

Figure S4. Full-length blots used in Figure 5b

Figure S5. Full-length blots used in Figure 5d

Figure S6. LC-MS/MS spectra and LC-chromatograms of RvD2 and RvD2-d5 internal standard

Figure S7. Full-length blots used in Figure 6

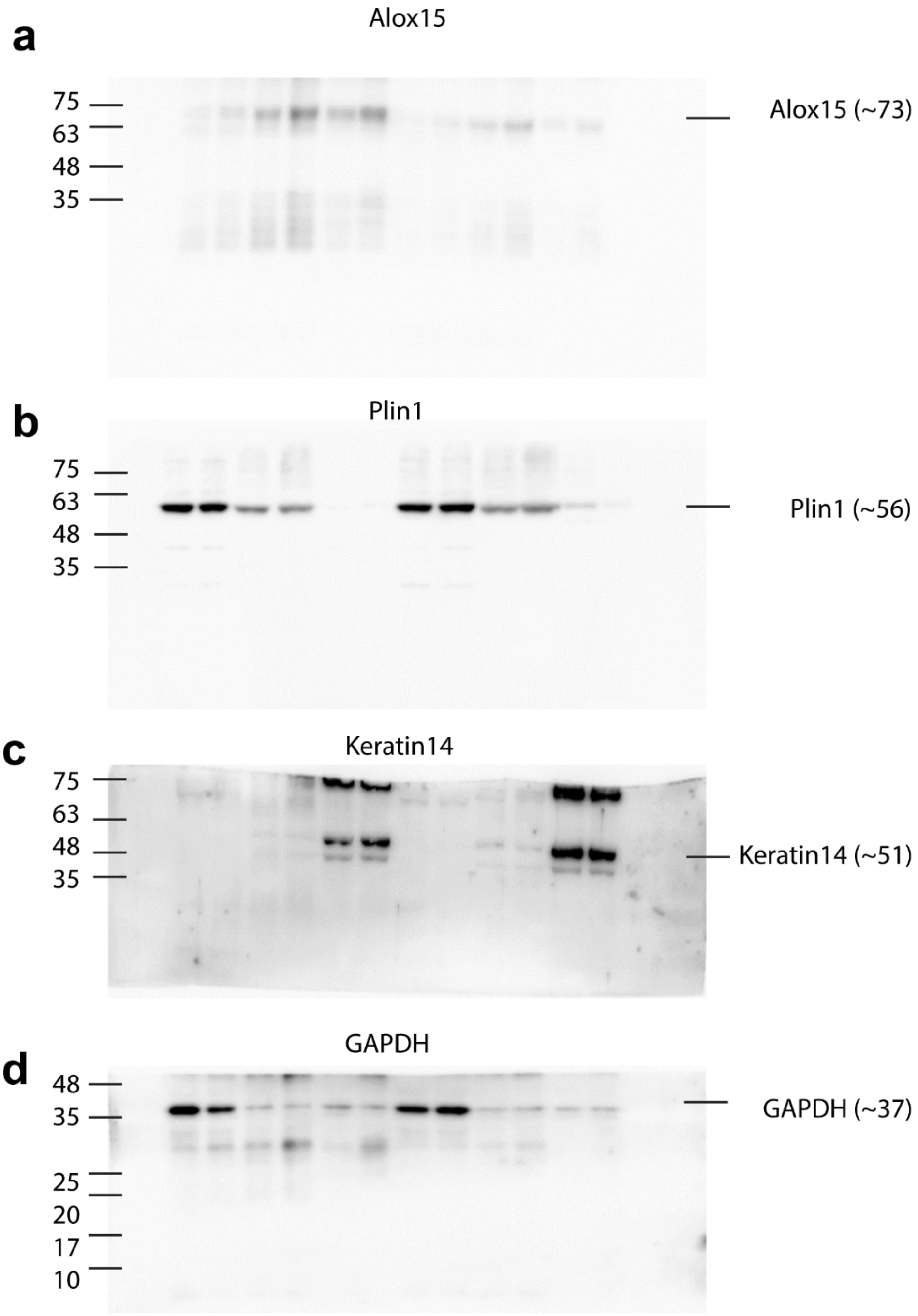

Figure S1. Full-length blots used in Figure 1

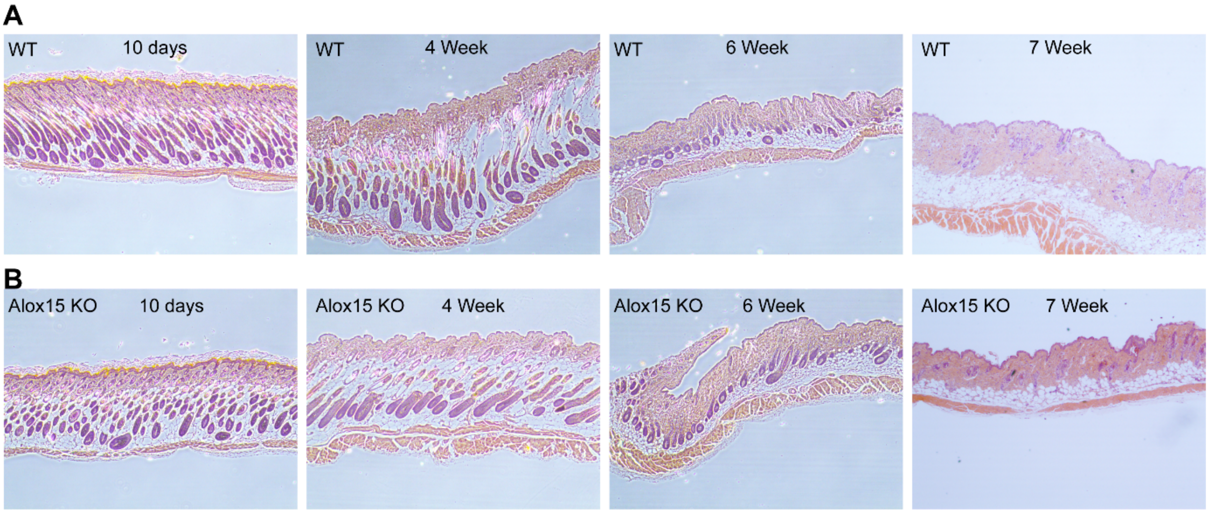

Figure S2. Histological analysis of the dorsal skin of Alox15 KO mice and wild type mice between 10 days to 7 weeks. The skin morphogenesis (i.e. 10 days: anagen), and the first hair cycle (i.e. 4 weeks: anagen, 6 weeks: catagen, 7 weeks: telogen) in Alox15 KO mice demonstrated similar pattern with wild type mice.

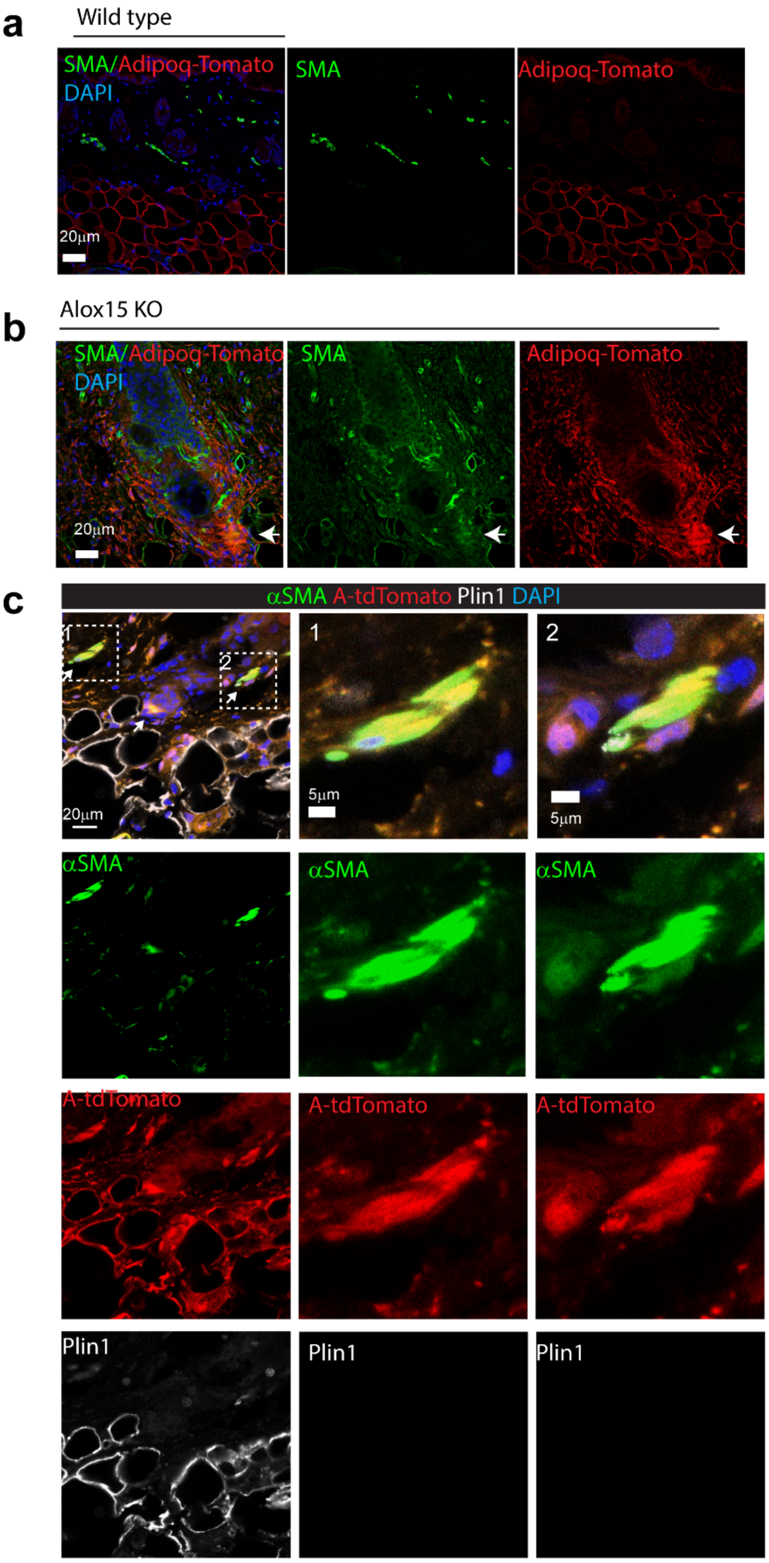

Figure S3. Lineage tracing study of Alox15KO-AdipoqCre-tdTomato mice related to Figure 4.

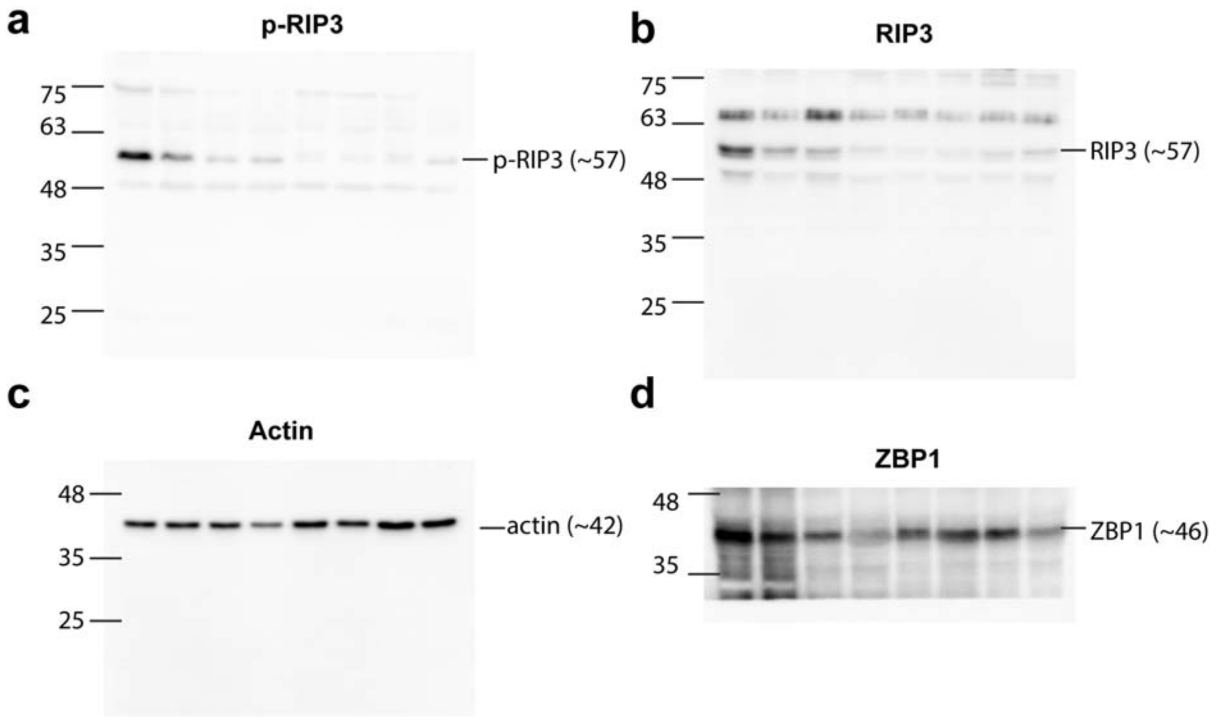

Figure S4. Full-length blots used in Figure 5b

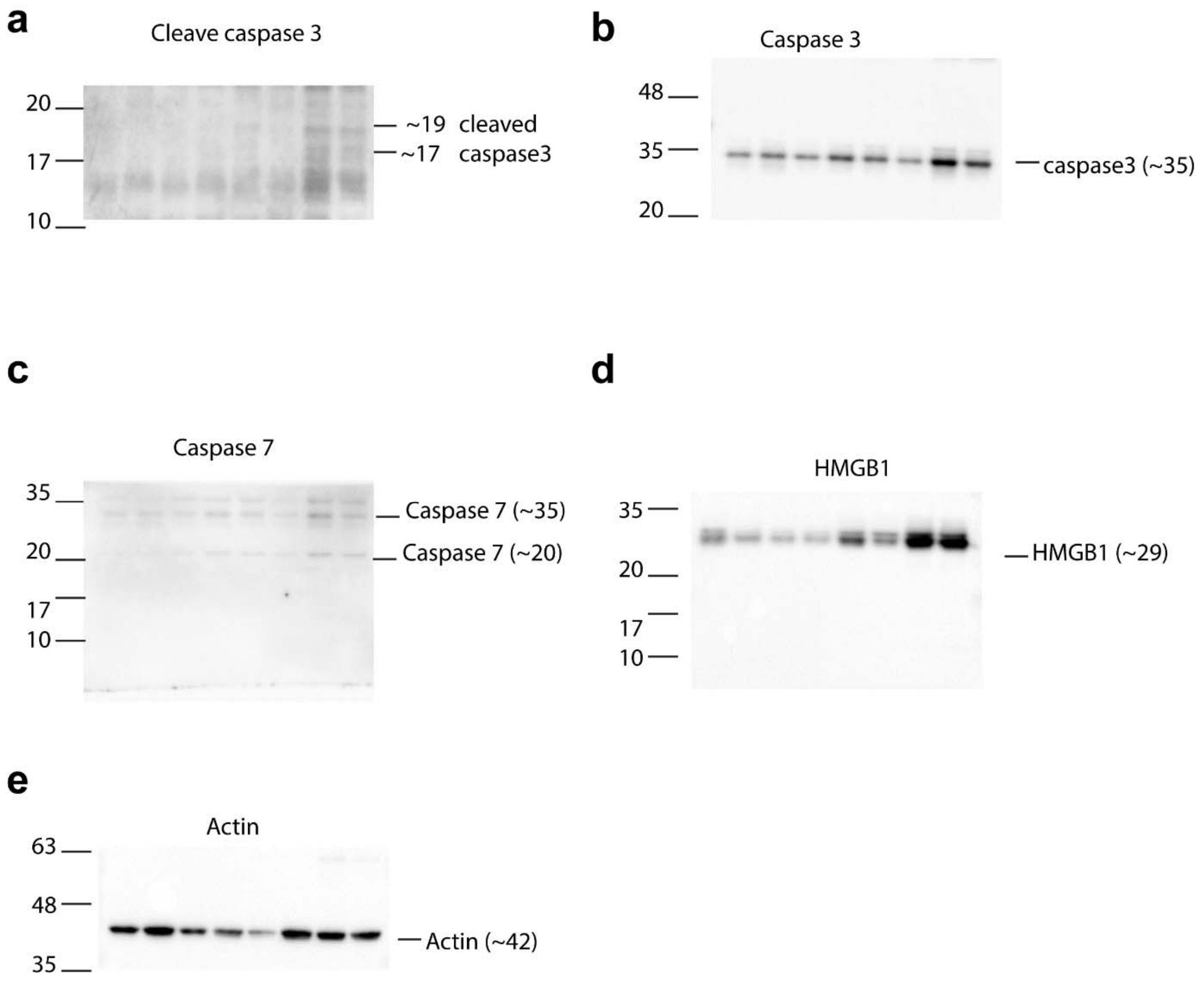

Figure S5. Full-length blots used in Figure 5d

RvD2 detected in Tissue

Chromatogram

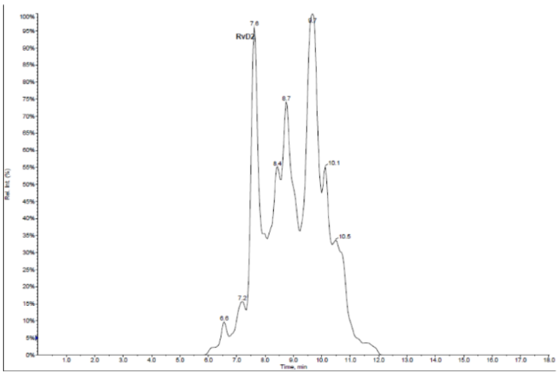

EPI spectrum

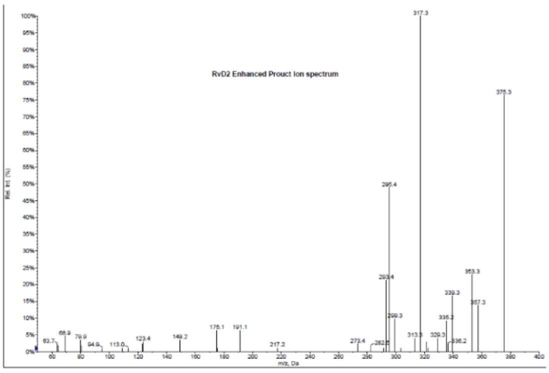

RvD2-d5 internal standard

Chromatogram

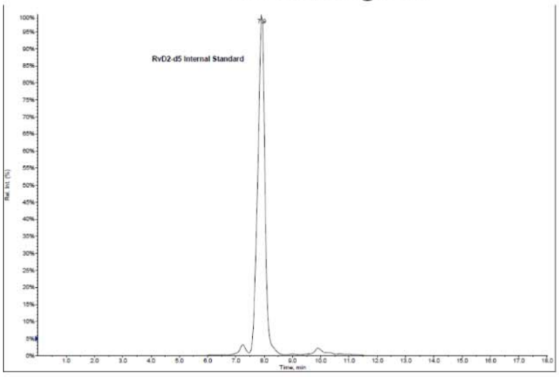

EPI spectrum

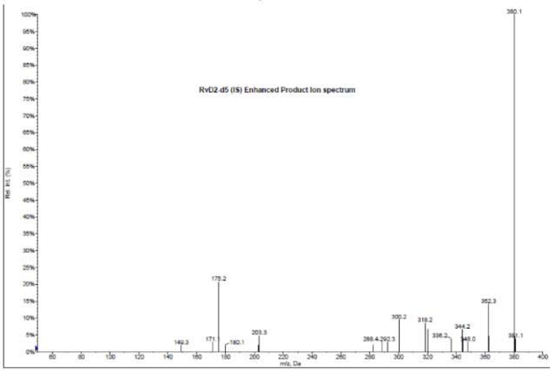

Figure S6. LC-MS/MS spectra and LC-chromatograms of RvD2 and RvD2-d5 internal standard

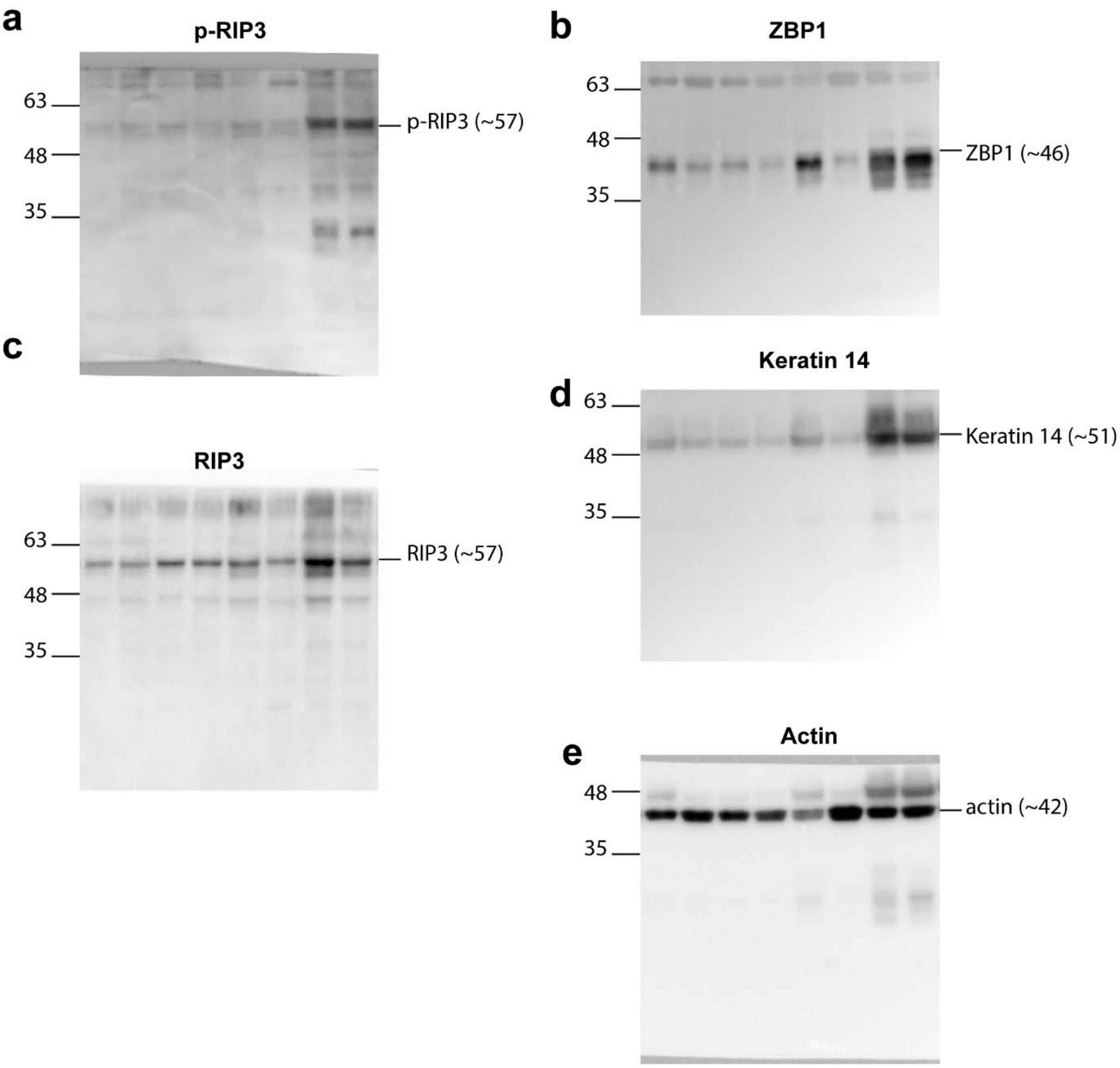

Figure S7. Full-length blots used in Figure 6
